# Determinants of early antenatal care visits among women of reproductive age in Ghana: evidence from the recent Maternal Health Survey

**DOI:** 10.1186/s12884-024-06490-3

**Published:** 2024-04-24

**Authors:** Aaron Asibi Abuosi, Emmanuel Anongeba Anaba, Anita Anima Daniels, Anita Asiwome Adzo Baku, James Akazili

**Affiliations:** 1 Department of Health Services Management, Business School, University of Ghana, Legon-Accra, Ghana; 2 Department of Population, Family and Reproductive Health, School of Public Health, University of Ghana, Legon-Accra, Ghana; 3 School of Public Health, C.K. Tedam University of Technology and Applied Sciences, Navrongo, Ghana; 4 BCEPS, University of Bergen, Bergen, Norway

**Keywords:** Antenatal care, Women of reproductive age, Maternal health survey, Determinants, Ghana

## Abstract

**Background:**

Antenatal care services play a crucial role in promoting positive pregnancy outcomes by facilitating the early identification of pregnancy risk factors and early diagnosis of pregnancy-related complications. This study aimed to assess the frequency and timing of ANC attendance of mothers in Ghana as well as determine the predictors of early ANC attendance.

**Methods:**

The data for this study was extracted from the 2017 Ghana Maternal Health Survey (GMHS). The study population was women aged 15–49 years with a live birth or stillbirth in the 5 years preceding the survey. Data was analysed using STATA/SE version 17, using descriptive statistics and multiple binary logistic regression analysis.

**Results:**

It was found that 44.4% of the women obtained eight (8) + ANC visits. A majority of the women (66%) initiated ANC visits in the first trimester of pregnancy. Early ANC visit was significantly associated with age of the respondent, education, wealth index, religion, region and reason for first ANC visit. For instance, women between the ages of 25–29 years (aOR = 1.75, 95% CI: 1.31–2.33) had increased odds of early ANC visit compared to those aged 15–19 years. Women with higher education (aOR = 1.83, 95% CI: 1.27–2.64) were about twice as likely to initiate early ANC visits compared to those with no education. Also, women in the highest wealth index (aOR = 2.43, 95% CI: 1.83–3.23) were two times more likely to initiate early ANC visits compared to those in the lowest wealth index.

**Conclusion:**

This study has shown that a majority of women in Ghana start their first ANC visit during the first trimester of pregnancy. A considerable proportion of the women failed to meet the WHO’s recommendation of having a minimum of eight ANC visits throughout pregnancy. Early ANC visit was determined by socio-demographic factors. Going forward, it should be a priority for stakeholders to ensure that ANC services are accessible to all mothers in a timely manner.

## Background

Antenatal care (ANC) is the care given to pregnant women so that they have safe pregnancy and healthy baby [1]. The World Health Organization recommends a minimum of eight antenatal care visits, initiating ANC during first trimester, giving birth in facilities, and postnatal care within 24 h of birth to reduce maternal and perinatal mortality [2, 3]. The provision of ANC services has a positive impact on pregnancy as it helps in the early identification of pregnancy risk factors and early diagnosis of complications in pregnancy such as preterm delivery [4]. The positive impact can be achieved through screening for pregnancy problems, assessing pregnancy risk, treating problems that may arise during the antenatal period, giving medication that may improve pregnancy outcomes, providing information to the pregnant woman, preparing physically and psychologically for childbirth and parenthood [5].

A number of studies have identified the lack of antenatal care as a risk factor for maternal morbidity and mortality [6–8]. Since inadequate ANC is associated with poor pregnancy outcomes, it is vital for health policymakers to better understand the factors influencing optimum utilization of ANC services. Utilization of ANC services during pregnancy will lead to further utilization of additional maternal services like institutional delivery and seeking assistance for complications during delivery and postnatal period [9].

Few studies have been done in developing countries to examine factors affecting early ANC attendance. In Ethiopia [10], reported that only 117 (26.2%) pregnant mothers started their first ANC visit early. Mothers with no parity before, had good knowledge on early ANC and planned pregnancy were significantly associated with early ANC visit. In South Africa [11], , revealed that 51% of rural women and 28% of peri-urban women presented late for first ANC. Rural women were more likely to present late for first ANC visit and report barriers to accessing ANC services. Late ANC presentation in rural communities was associated with being married, employed, less than 20 years of age, and reporting an unplanned pregnancy. Late presentation in peri-urban communities was associated with unplanned pregnancy, being told to come back later to initiate ANC after presenting early and being pregnant for the first time.

In Ghana, although attendance of at least one ANC visit is nearly universal and 89% of women reported having 4 or more ANC visits, only 64% of women follow the recommendation of starting ANC in the first trimester. Facility deliveries continue to hover around 57–79% [12–14]. Anecdotal reports suggest that these figures may be overestimates of ANC attendance, as bringing women to ANC early remains a major challenge. Many women start ANC late, do not have the required number of visits, and often have complications [15]. The remaining women who do not start ANC in first trimester and who do not receive adequate ANC visits are the hard to reach population [16].

The most commonly cited reasons for not seeking maternal health services include lack of money, the perception that obstetric care is not necessary, and transportation problems [13]. In order to encourage women to seek maternal services at health facilities, the government of Ghana has waived fees at public facilities starting in 2004 [17]. Yet a recent assessment revealed that the Free Maternal Health Care Policy (FMHCP) exists only on paper and many women have to make payments for ANC and skilled delivery services [13]. Despite the limited studies on factors affecting early ANC attendance in developing countries, to the best of the author’s knowledge, no study has been done in Ghana to examine this nagging problem. This study therefore seeks to fill this gap. The objectives of this study are to assess the frequency and timing of ANC attendance of mothers in Ghana as well as determine the predictors of early ANC visits.

## Methods

### Study design and data source

The data for this study was extracted from the 2017 Ghana Maternal Health Survey (GMHS). The survey was conducted by Ghana Statistical Service (GSS) with technical support from Inner City Fund (ICF) through the Demographic and Health Survey (DHS) program. GMHS used a multi-stage sampling where the first stage involved the selection of enumeration areas with probability proportional to the sizes of enumeration areas. In the second stage, households were selected from each enumeration area using systematic random sampling. Details of the sampling procedure is publicly available [18].The 2017 GMHS was conducted among women aged 15–49 years who delivered a live birth or stillbirth from the period between 2012 and 2017.

The study population were women aged 15–49 years with a live birth or stillbirth in the 5 years preceding the survey who received antenatal care from a skilled provider (doctor, nurse/midwife, or community health officer/nurse) during their most recent pregnancy. Women who did not receive ANC or received ANC from traditional birth attendants, were excluded from this study. The GMHS interviewed 25,062 women (unweighted). This study focused on women with data on ANC visits, hence a total of 13,215 women (unweighted) were excluded from the analysis. Therefore, a sample of 11,847 (unweighted) and 11,291 (weighted) women aged 15–49 years were included in this analysis.

### Measurement

The outcome variable of interest was ANC attendance, a dummy variable coded 1 = early ANC visit (within the first three months of pregnancy); and 0 = otherwise. The primary independent variables of interest were all categorical variables, including getting permission to attend ANC; getting money to attend ANC; payment for ANC services; and problem during first ANC visit. The secondary independent variables of interest included health insurance status; distance to health facility; region of respondent; religion; age; educational level; wealth quintile; and type of place of residence, all categorical variables. Detail about the coding is provided elsewhere [18].

### Data analysis

Data was analysed using STATA/SE version 17. At the univariate level, descriptive statistics including frequencies and percentages were analysed for the respective variables. At the multivariable level, binary logistic regression analysis, both crude and adjusted analyses, were employed to assess the relationship between independent variables and early ANC visit. The crude and adjusted odds ratios were estimated at 95% confidence interval (CI) and significance level of 0.05. The “svyset” feature in STATA 17 was used with the weighting, cluster and strata variables. The survey protocol for GMHS was reviewed and approved by the ICF Institutional Review Board. This study analyzed data from GMHS, therefore, ethical approval was not required. We received permission from the DHS Program to use the data.

## Results

### Descriptive statistics

The results showed that one-fifth (23.9%) of the participants were age 25–29 years and 30–34 years (23.6%). Four in ten (40.2%) participants had completed junior high school and one-fifth (20.7%) of them were in the poorest wealth index. Seven in ten (77.7%) participants professed Christianity and majority (50.9%) of the participants resided in rural areas. In addition, more than half (57.8%) of the participants had health insurance. Exactly 44.6% of the participants paid for ANC services, and 86% of the participants made their first ANC visit for a checkup. A majority of the participants did not perceive distance to a health facility (74.6%) and getting permission to seek care (93.9%) as barriers to seeking care. A little above half of the participants (50.8%) did not face difficulties in getting money for treatment. Regarding the frequency of ANC visits, 47.5% of the participants made between four to seven visits and 44.4% made eight + visits. A majority (66%) of the participants started ANC visit in the first trimester of pregnancy (Table 1).

**Table 1:** Participant characteristics

Characteristic (*n* = 11,291)	n	%
**Age of respondent (years)**		
15–19 20–24 25–29 30–34 35–39 40–44 45–49	560 1978 2700 2668 2045 983 357	5.0 17.5 23.9 23.6 18.1 8.7 3.2
**Educational level**		
No education Primary Junior high Senior high Higher	2641 1986 4536 1415 713	23.4 17.6 40.2 12.5 6.3
**Wealth index**		
Lowest Second Middle Fourth Highest	2333 2408 2264 2252 2034	20.7 21.3 20.1 19.9 18.0
**Religion**		
Christianity Muslim Traditional No religion	8779 1958 242 313	77.7 17.3 2.1 2.8
**Place of residence**		
Urban Rural	5542 5749	49.1 50.9
**Region**		
Western Central Greater Accra Volta Eastern Ashanti Brong Ahafo Northern Upper East Upper West	1441 1040 1693 909 1130 2080 1179 1063 443 314	12.8 9.2 15.0 8.0 10.0 18.4 10.4 9.4 3.9 2.8
**Covered by health insurance**		
Yes No	5783 4215	57.8 42.2
**Reason for first ANC visit**		
Problem No problem (Just for a checkup)	1579 9712	14.0 86.0
**Payed for ANC services**		
Yes No	4396 5468	44.6 55.4
**Distance to health facility**		
Big problem Not a big problem	2873 8418	25.4 74.6
**Getting permission**		
Big problem Not a big problem	691 10,601	6.1 93.9
**Getting money for treatment**		
Big problem Not a big problem	5557 5734	49.2 50.8
**Frequency of ANC visits**		
1–3 visits 4–7 visits 8 + visits	921 5348 4998	8.2 47.5 44.4
**Timing of ANC visits**		
0–3 months (early) 4 + months (late)	7443 3829	66.0 34.0

### Factors associated with ANC visits among women in Ghana

The crude analysis showed that early ANC visit was significantly associated with age of the respondent, educational level, wealth index, religion, type of place of residence, geographical region, reason for first ANC visit, distance to a health facility, getting permission for treatment and getting money for treatment. For instance, women aged 30–34 years (cOR = 2.17, 95% CI: 1.65–2.84) were more likely to initiate early ANC visits compared to adolescent girls. Women with higher education (cOR = 2.54, 95% CI: 2.11–3.06) were 2.5 times more likely to initiate early ANC visits compared to those with no education. Compared to women in the lowest wealth index, those in the highest wealth index had increased odds of early ANC visit (cOR = 2.54, 95% CI: 2.11–3.06). Women who professed Islam (cOR = 0.80, 95% CI: 0.71–0.90) had decreased odds of early ANC visit compared to those who professed Christianity. Women in rural areas (cOR = 0.85, 95% CI: 0.75–0.96) were less likely to initiate early ANC visit compared to those in urban areas. Surprisingly, women in the Upper West region (cOR = 1.38, 95% CI: 1.09–1.74) had increased odds of early ANC visit compared to those in the Greater Accra region. Women who did not have a problem with distance to a health facility (cOR = 1.17, 95% CI: 1.03–1.32), getting permission for treatment (cOR = 1.22, 95% CI: 1.00-1.49) and getting money for treatment (cOR = 1.29, 95% CI: 1.17–1.43) had increased odds of early ANC visit compared to their counterparts (Table 2).

In the adjusted analysis, early ANC visit was significantly associated with age of the respondent, education, wealth index, religion, region and reason for first ANC visit. For example, women between the ages of 25–29 years (aOR = 1.75, 95% CI: 1.31–2.33) had increased odds of early ANC visit compared to those aged 15–19 years. Women with higher education (aOR = 1.83, 95% CI: 1.27–2.64) were about twice more likely to initiate early ANC visit compared to those with no education. Also, women in the highest wealth index (aOR = 2.43, 95% CI: 1.83–3.23) were two timely more likely to initiate early ANC visit compared to those in the lowest wealth index. Women who professed Islam (aOR: 0.83, 95% CI: 0.71–0.97) had decreased odds of initiating early ANC visit compared to those who professed Christianity. Interestingly, women in the Upper West region (aOR = 2.31, 95% CI: 1.70–3.15) had increased odds of early ANC visit compared to those in the Greater Accra region. Women who went with no reported problem but just to check up on themselves and their babies for their first ANC visit had reduced odds of early ANC visit compared to their counterparts (Table 2).

**Table 2:** Binary logistic regression analysis of factors associated with early ANC visits

Characteristic	cOR (95% CI)	*p*-value	aOR (95%CI)	*p*-value
**Age of respondent (years)**				
15–19 20–24 25–29 30–34 35–39 40–44 45–49	1(ref) 1.61(1.21–2.13) 2.10(1.59–2.75) 2.17(1.65–2.84) 2.00(1.50–2.66) 1.81(1.34–2.45) 1.48(1.02–2.13)	0.001 0.000 0.000 0.000 0.000 0.036	1(ref) 1.51(1.12–2.05) 1.75(1.31–2.33) 1.71(1.28–2.29) 1.67(1.24–2.26) 1.60(1.15–2.22) 1.31(0.89–1.93)	0.007 0.000 0.000 0.001 0.004 0.165
**Educational level**				
No education	1(ref)		1(ref)	
Primary Junior high Senior high Higher	1.03(0.89–1.18) 1.25(1.10–1.41) 1.48(1.23–1.77) 3.08(2.32–4.09)	0.653 0.000 0.000 0.000	1.02(0.86–1.21) 1.12(0.95–1.33) 1.12(0.89–1.41) 1.83(1.27–2.64)	0.778 0.150 0.316 0.001
**Wealth index**				
Lowest	1(ref)		1(ref)	
Second Middle Fourth Highest	1.03(0.89–1.20) 1.15(0.99–1.33) 1.39(1.18–1.63) 2.54(2.11–3.06)	0.641 0.053 0.000 0.000	1.05(0.89–1.24) 1.18(0.97–1.43) 1.44(1.15–1.79) 2.43(1.83–3.23)	0.543 0.082 0.001 0.000
**Religion**				
Christianity Muslim Traditional No religion	1(ref) 0.80(0.71–0.90) 0.60(0.44–0.82) 0.77(0.59–1.02)	0.000 0.002 0.070	1(ref) 0.83(0.71–0.97) 0.80(0.51–1.25) 0.92(0.67–1.26)	0.020 0.336 0.625
**Place of residence**				
Urban Rural	1(ref) 0.85(0.75–0.96)	0.010	1(ref) 1.11(0.95–1.30)	0.152
**Region**				
Western Central Greater Accra Volta Eastern Ashanti Brong Ahafo Northern Upper East Upper West	0.90(0.69–1.18) 1.01(0.77–1.31) 1(ref) 1.00(0.76–1.31) 1.20(0.95–1.51) 1.00(0.80–1.24) 0.90(0.71–1.14) 0.69(0.54–0.88) 1.29(0.99–1.67) 1.38(1.09–1.74)	0.479 0.926 0.997 0.116 0.990 0.412 0.003 0.053 0.006	1.26(0.92–1.72) 1.46(1.10–1.93) 1(ref) 1.51(1.05–2.19) 1.85(1.40–2.45) 1.27(0.97–1.66) 1.38(0.96–1.83) 1.33(0.96–1.83) 2.43(1.76–3.37) 2.31(1.70–3.15)	0.143 0.008 0.025 0.000 0.071 0.027 0.079 0.000 0.000
**Covered by health insurance**				
Yes No	1(ref) 0.92(0.82–1.02)	0.129	1(ref) 1.00(0.88–1.12)	0.990
**Reason for first ANC visit**				
Problem No problem (Just for a checkup)	1(ref) 0.53(0.46–0.63)	0.000	1(ref) 0.52(0.44–0.63)	0.000
**Payed for ANC services**				
Yes No	1(ref) 1.09(0.98–1.21)	0.099	1(ref) 1.12(0.99–1.27)	0.062
**Distance to health facility**				
Big problem Not a big problem	1(ref) 1.17(1.03–1.32)	0.010	1(ref) 0.96(0.82–1.13)	0.654
**Getting permission**				
Big problem Not a big problem	1(ref) 1.22(1.00-1.49)	0.042	1(ref) 1.11(0.87–1.42)	0.389
**Getting money for treatment**				
Big problem	1(ref)		1(ref)	
Not a big problem	1.29(1.17–1.43)	0.000	1.03(0.91–1.17)	0.586

## Discussion

This study aimed to assess the frequency and timing of ANC visit among women in Ghana as well as determine the predictors of early ANC visits. The results showed that less than half of the women obtained eight + ANC visits. This finding supports evidence from previous observations in sub-Sahara Africa [19]. For example, a study that analyzed the 2019 Ghana Malaria Indicator Cluster Survey data showed that four in ten women made eight + ANC visits [20]. Another study that analyzed the 2017–2018 Ghana Multiple Indicator Cluster Survey data found that about one-third of the women obtained eight + ANC visits [21]. However, the prevalence of eight + ANC visits in Ghana is relatively higher than findings in other West Africa countries. A further analysis of the 2018 Nigeria DHS and the 2017/2018 Benin DHS found that 17.4% and 8% of women obtained eight + ANC visits respectively [19, 22].

Regarding the timing of ANC visit, it was found that six in ten (66%) women started ANC visit in the first trimester of pregnancy. This finding is consistent with the finding of a previous study [20] who also found that 68% of women in the country started ANC in the first trimester. However, a further analysis of the Ethiopian DHS revealed that 32.7% of women initiated early ANC visit [23]. Similarly, the Myanmar DHS revealed that 47% of the women started ANC visit in the first trimester of pregnancy [24].

Possible explanations for the difference in findings include the Free Maternal Health Care Policy (FMHCP) and National Health Insurance Scheme (NHIS) in Ghana. In 2008, Ghana implemented a FMHCP which is a vital component of the NHIS. This policy ensures that all pregnant women are exempted from paying NHIS premiums when they subscribe or renew their membership. Under this policy, expectant mothers are entitled to a wide range of medical services that fall under NHIS coverage, including antenatal, delivery, and postnatal care, as well as neonatal care for infants for the first three months after birth [25]. There is evidence to show that the FMHCP and NHIS have contributed significantly to improving utilization of maternal healthcare services in Ghana [26]. A study found that women with NHIS membership were about forty times more likely to access adequate ANC services compared to their counterparts [25]. In addition, another study revealed that a majority of women reported that NHIS is increasing access to maternal healthcare services [27].

A further analysis showed that adult women were more likely to initiate early ANC visits compared to adolescent girls. This finding is expected and consistent with evidence from previous studies in sub-Sahara Africa [28]. A number of factors may explain this observation. Firstly, adolescent girls might have inadequate knowledge about the timing for ANC visit. Hence, they are less likely to know the right period to initiate ANC visit. There is evidence to show that women with knowledge of ANC timing had increased odds of early ANC visit [29]. Secondly, most adolescent pregnancies may be unwanted or unplanned. Therefore, they may hesitate to inform their parents or healthcare providers of their pregnancy, which can result in delayed initiation of ANC visits. A study showed that women who wanted a pregnancy had increase odds of early ANC visit compared to their counterparts [29]. These findings have implications for maternal and newborns health outcomes. Adolescent mothers are at a higher risk of pregnancy-related complications [30]. Additionally, children born to adolescent mothers are at a higher risk of low birth weight and severe neonatal conditions [31]. Hence, untimely initiation of ANC visit may put them at risk of complications during delivery and adverse birth outcomes.

Other expected findings were that women with higher education and those with higher socio-economic status had increased odds of early ANC visit. These findings confirm the observations of previous investigations on timing of ANC visits [28].These relationships may be partly explained by the fact that women with higher education are literate and can easily access information about early ANC services. In addition, women with higher education and higher socio-economic status are more likely to afford the expenses associated with accessing ANC services, including transportation cost and ANC service charges. It was also not surprising to observe that women who had problems with their pregnancy were more likely to initiate early ANC visit. This is because pregnancy-related problems may create a need for healthcare services, leading to early ANC visit.

Perhaps the most striking finding is that women in poor geographical regions such as Upper East and Upper West regions, were twice more likely to initiate early ANC visit compared to those in the capital city of Ghana. It is difficult to explain this result, but it might be attributed to a number of factors. First, the Upper East region has the highest ANC coverage and the Upper West region has the highest NHIS coverage in the country [18]. Health insurance coverage may increase financial access to health care services, especially under the FMHCP where NHIS subscribers have access to free maternal healthcare services. Further research that thoroughly investigate this finding can help give a comprehensive understanding.

The findings suggest that a significant proportion of mothers in Ghana failed to adhere to the World Health Organization’s 2016 recommendation of a minimum of eight antenatal care visits, as well as initiate ANC in the first trimester of pregnancy. Among other factors, the suboptimal use and late initiation of ANC visits may be due to financial constraints. Evidence from this study showed that four in ten women had difficulty in getting money for treatment or were not covered by health insurance. In addition, some women reported that they paid for ANC services. These challenges may pose as financial barriers to accessing ANC services.

These findings have implications for maternal and child health outcomes. For instance, inadequate ANC visit and late initiation of ANC visit may increase the risk of maternal and newborn deaths [32]. In the quest to achieve Sustainable Development Goal of reducing maternal mortality to less than 70 per 100,000 live births by 2030 [33], Ghana Health Service, the Ministry of Health and the National Health Insurance Authority should take proactive measures to improve ANC coverage in the country. It is a matter of concern that some mothers pay for ANC services, despite the fact that such services are intended to be provided free of charge under the FMCHP. This issue must be addressed by stakeholders in a timely and effective manner. In addition, maternal health education interventions should target adolescent girls, women with no formal education as well as those from poor socio-economic background. Also, it is important to prioritize early ANC visits among women who do not have pregnancy-related problems.

### Strengths and limitation of the study

A major strength of this study is the use of nationally representative data. Also, the survey employed robust sampling techniques to recruit the participants as well employed standard instruments and trained enumerators to collect the data. The rigor in the methods has improved the validity and reliability of the findings. The findings of this study will be relevant for developing national policies regarding antenatal care services. The major limitation of this study is the absence of qualitative data exploring reasons for early or late ANC attendance to triangulate the quantitative findings.

## Conclusion

This study has shown that a majority of women in Ghana start their first ANC visit during the first trimester of pregnancy. However, a considerable proportion of them failed to meet the WHO’s recommendation of having eight or more ANC visits throughout pregnancy. The factors that influenced early initiation of ANC visits were being an adult woman, having higher education, having a higher socio-economic status, being a Christian, living in poor geographical regions, and having pregnancy problems. Future studies should investigate the reasons behind early or late ANC visits. Going forward, it should be a priority for stakeholders to ensure that ANC services are accessible to all mothers in a timely manner. The findings of this study have significant implications for future maternal health policies and programmes.

## Data Availability

The data used in this study is owned by The DHS Program, therefore, the authors cannot share the data. Interested persons can contact The DHS Program for the data (https://dhsprogram.com/data/available-datasets.cfm). The authors confirm they did not have any special access or privileges to the data that other researchers would not have.

## Abbreviations

GMHS: Ghana Maternal Health Survey
ANC: Antenatal care
NHIS: National Health Insurance Scheme
SSA: Sub-Sahara Africa
FMHCP: Free Maternal Health Care Policy
WHO: World Health Organization
